# Shared dynamics of LeuT superfamily members and allosteric differentiation by structural irregularities and multimerization

**DOI:** 10.1098/rstb.2017.0177

**Published:** 2018-05-07

**Authors:** Luca Ponzoni, She Zhang, Mary Hongying Cheng, Ivet Bahar

**Affiliations:** Department of Computational and Systems Biology, School of Medicine, University of Pittsburgh, Pittsburgh, PA 15213, USA

**Keywords:** LeuT-fold, secondary transporters, elastic network models, intrinsic dynamics, alternating access, conformational changes

## Abstract

The LeuT-fold superfamily includes secondary active transporters from different functional families, which share a common tertiary structure, despite having a remarkably low sequence similarity. By identifying the common structural and dynamical features upon principal component analysis of a comprehensive ensemble of 90 experimentally resolved structures and anisotropic network model evaluation of collective motions, we provide a unified point of view for understanding the reasons why this particular fold has been selected by evolution to accomplish such a broad spectrum of functions. The parallel identification of conserved sequence features, localized at specific sites of transmembrane helices, sheds light on the role of broken helices (TM1 and TM6 in LeuT) in promoting ion/substrate binding and allosteric interconversion between the outward- and inward-facing conformations of transporters. Finally, the determination of the dynamics landscape for the structural ensemble provides a promising framework for the classification of transporters based on their dynamics, and the characterization of the collective movements that favour multimerization.

This article is part of a discussion meeting issue ‘Allostery and molecular machines’.

## 1. Introduction

**Figure 1.**
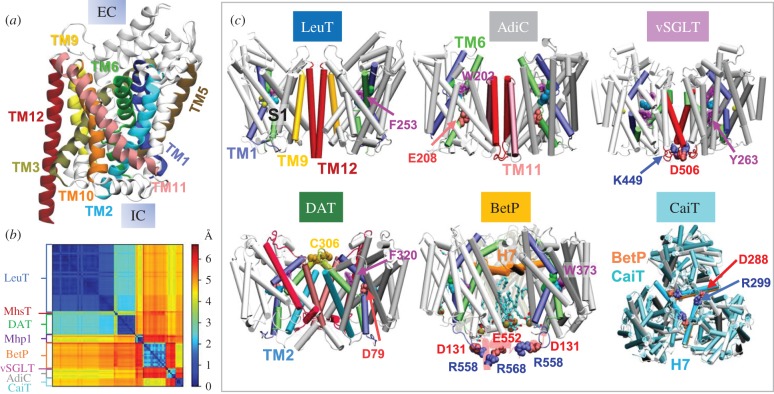
LeuT-fold shared by monomeric, dimeric and multimeric transporters. (*a*) LeuT-fold. Selected helices are colour-coded and labelled, using conventional numbering [3]. (*b*) RMSDs between structurally resolved LeuT-fold monomers/protomers (see electronic supplementary material, table S1). (*c*) Different oligomerization states of LeuT-fold protomers, illustrated for LeuT dimer (PDB: 2A65), AdiC dimer (PDB: 3L1L), vSGLT dimer (PDB: 3DH4), enhanced by interfacial salt-bridge K449-D506, hDAT dimer model [4] and two trimeric forms, BetP (PDB: 4C7R) and CaiT (PDB: 4M8J). *Side views* shown in all panels, except for the last where *top view* of CaiT structurally aligned against BetP is shown. Broken helices TM1 and TM6 are coloured in *light blue* and *green*; substrate and sodium ions are in *cyan* and *yellow van der Waals *(vdW)* spheres,* respectively. A number of functional residues are shown in *vdW spheres*: Gly (*green*) at the substrate-binding site, aromatic EC gating residues (*purple*), and positively (*blue*) or negatively (*light red*) charged residues participating in substrate binding or IC salt-bridge formation. Anionic lipids bound to the BetP trimer are shown in *cyan thick-stick* representation, and phosphorus atoms, as *tan spheres*. H7 helices forming the trimeric interface are shown in BetP (*orange cylinders*) and CaiT (*cyan cylinders*), along with the interfacial salt-bridge R299-D288. See also figure 2, and the superposition of the transporters in the OFS and IFS in electronic supplementary material, figure S1.

Secondary active transporters translocate small molecules such as neurotransmitters, nutrients and metabolites across cellular membranes, using the energy provided by the co-transport (symport) or exchange (antiport) of ions or other solutes down their electrochemical gradients. Remarkably, several secondary active transporters, though belonging to genetically and functionally distant families, share a common architecture (or fold). Four common folds among transporters are the LeuT-, MFS-, Glt_Ph_- and NhaA-folds [1,2]. Prototypical proteins, first-resolved in each case, are: the bacterial (*Aquifex aeolicus*) leucine transporter (figure 1*a*), a member of the family of neurotransmitter : sodium symporters (NSSs) [5]; a human glucose transporter belonging to the major facilitator superfamily [6]; the archaeal aspartate transporter, Glt_Ph_, from *Pyrococcus horikoshii* [7], which has broadly served as a structural model for human excitatory amino acid transporters; and the Na^+^/H^+^ antiporter, NhaA, from *Escherichia coli* [8].

An immediate question concerning the selection of a small pool of folds by a large number of transporters involved in different functions, and vastly differing in their sequence, is what is special about those folds that lend themselves to different functionalities. What are their structural and dynamic characteristics that are exploited, or how do they adapt to different functions? Differences can be at various levels, from sequence, to structural motifs, or quaternary organization (for multimeric transporters) while maintaining the tertiary fold. We will focus here on the LeuT-fold superfamily, which probably has the broadest representation in the Protein Data Bank (PDB) among the four folds. LeuT has served as a model for exploring the mechanism of action of monoamine transporters such as the dopamine transporter (DAT) [9] or the serotonin transporter (SERT).

A classical model for the transport mechanism of secondary transporters is the alternating access model [10,11]: mainly, the transporter undergoes a structural change from the outward-facing state (OFS) for substrate/ion uptake from the extracellular (EC) medium, to the inward-facing state (IFS) for release of its cargo to the intracellular (IC) medium, and *vice versa* to resume the transport cycle. While this model has helped appreciate the mechanistic aspects of substrate transport, recent structural data integrated with biochemical and computational studies have improved our understanding of the complex machinery of transporters [1,2,12–15]. These studies led to the definition of rocking bundle for the LeuT-fold [16] or rock-switch mechanism for the MFS-fold [17]. In the case of glutamate transporters, an elevator-like sliding of the transport domains [18,19] emerged, shared with Na^+^/proton antiporters or other Na^+^/dicarboxylate co-transporters [20]. In addition to these global motions, local EC/IC gating events, often enabled by the side chain isomerization or reorientation of selected amino acids or small motifs such as helical hairpins, have been elucidated.

More importantly, couplings between global and local events [2,21] or even the protonation state of residues [22,23] have been reported to direct substrate translocation. In LeuT, binding of substrate from one side drives the closure of the ‘thin’ (EC) gate, the repacking of transmembrane (TM) helices and the opening of the ‘thick gate’ [12], leading to the release of substrate to the other side [24,25]; and the hydration of the inward-facing (IF) vestibule due to the migration of a co-transported Na^+^ ion (Na2) has been observed *in silico* [25–29] and suggested experimentally [30] to cooperatively stabilize LeuT IFS. Coupled ion binding and structural transitions have been reported for Glt_Ph_ [31]; and those between EC/IC gates and TM helical configuration have been observed for a glucose transporter belonging to the SWEET family [32]. Such couplings suggest an allosteric regulation of transport activity [2,33,34], although a systematic quantitative analysis of collective dynamics has not been carried out across all (or representative) members of a given fold family.

Another interesting observation is that structurally homologous transporters function in various oligomerization states, as illustrated in figure 1*c* for a few members of the LeuT-fold family. Their protomers retain their fold (and access to OFS and IFS) (electronic supplementary material, figure S1), suggesting that this modular fold is used to perform transport functions regardless of the oligomerization state. A similar observation was made in the trimeric Glt_Ph_: the individual protomers exhibit different levels of exposure to either the EC or IC region, without interference from the trimerization scaffold [35]. Recent examination of DAT dynamics also suggests that the dimeric architecture may facilitate the OFS ↔ IFS transition [4,36]. Observations in other families also suggest that multimerization can modulate function: e.g. dimerization reduces the substrate-binding affinity of a nitrate transporter (MFS-fold) [37]; and a trimeric SWEET transporter shows an allosteric coupling between protomer structure and function [38]. Thus, oligomerization may have allosteric effects, beyond that of assembling protomers around a stable scaffold.

In the present study, we first examine the sequence and structure properties of LeuT superfamily members, and then proceed to their dynamics to determine a ‘signature’ mobility profile shared by sequentially and functionally dissimilar LeuT-fold transporters that allosterically engages all TM helices. We further examine the role of structural irregularities such as helical disruptions, and that of multimerization, in the differentiation or allosteric modulation of transport activities, and determine the dynamics landscape of a large ensemble of structures sharing the LeuT-fold, which indicates the collective motions that underlie the OFS ↔ IFS transition or the multimerization of LeuT-fold members. Our analysis sheds light into the ways transporters achieve functional differentiation, while efficiently recruiting the same fold whose modular dynamics is exploited.

## 2. Results and discussion

### (a) Materials

We consider a set of 90 structures with LeuT-fold deposited in the PDB, which belong to five functional families, listed in the electronic supplementary material, table S1. The set includes crystallographic structures resolved for eight different transporters: LeuT in different conformational states, DAT, and MhsT from the NSS family; galactose transporter (vSGLT) from the sodium/solute symporter (SSS) family; betaine transporter (BetP) and carnitine/betaine antiporter (CaiT) in multiple states from the betaine/choline/carnitine transporters (BCCT); benzylhydantoin (BH) transporter Mhp1 from the nucleobase/cation symport-1 (NCS1) family; and arginine/agmatine antiporter AdiC from the amino acid/polyamine/organocation (APC) family.

### (b) Sequence differences confer specificity while maintaining the fold

The LeuT-fold (figure 1*a* and electronic supplementary material, figure S1) is characterized by 10 TM helices, organized into two pseudo-symmetric inverted repeats, TM1–TM5 and TM6–TM10 [3]. The electronic supplementary material, figure S1 displays the superposition of the transporters resolved in the OFS (A) and the IFS (B), highlighting the common fold shared by the superfamily, as well as the distinctive packing of TM helices to expose the EC or IC vestibule in the OFS and IFS, respectively.

Structural alignments of the transporters listed in electronic supplementary material, table S1 reveal differences of up to 6.5 Å root-mean-square deviation (RMSD) between pairs of transporters (figure 1*b*). Mainly, the structures resolved for the same protein (e.g. LeuT) in different conformations exhibit RMSDs of approximately 2.0 Å in general; those *within* the same family (e.g. NSS members LeuT, DAT and MhsT; or BCCT members BetP and CaiT) differ by 3–4.5 Å; while across families (e.g. BCCT and APC family members BetP and AdiC, respectively; or BCCT and NCS1 members BetP and Mhp1) the RMSDs may exceed 6 Å. Thus, although all the transporters have the same fold, there is a hierarchy of structural differences, increasing with their functional differences.

Pairwise alignments of LeuT-fold family sequences (electronic supplementary material, figure S2A) confirm their low sequence identities. Pairs belonging to the same family, e.g. DAT–LeuT (NSS), BetP–CaiT (BCCT) or AdiC–ApcT (APC), exhibit sequence identities of 0.25 ± 0.03; across families, the identities drop to 0.15 ± 0.05 (electronic supplementary material, figure S2B). If we focus on TM1 and TM6, the sequence identities are much higher within families (electronic supplementary material, figure S2C–E) (e.g. 0.60 ± 0.24 for NSS members, and 0.42 ± 0.02 for BCCT members), whereas there is a major drop across families. For example, CaiT TM1 shows sequence identities of 0.06 ± 0.02 with respect to most transporters; see details in electronic supplementary material, tables S2 and S3. The strong conservation within families and low conservation across families strongly suggest that these helices play a role in defining the specificity of the LeuT-fold transporters.

### (c) Functional significance of broken helices

**Figure 2.**
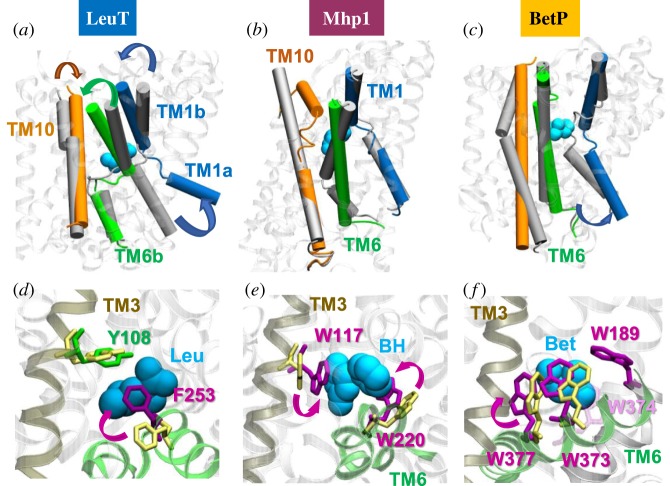
Structural and dynamic significance of helical disruptions. Changes in the orientations of TM1, TM6 and TM10 between the OFS (*grey*) and IFS (*coloured*) of (*a*) LeuT, (*b*) Mhp1 and (*c*) BetP. In the background, the OFS is displayed in *white ribbons*. Substrates are shown in *cyan spheres*. They bind near the disrupted regions of TM1 and TM6/TM10. (*d*–*f*) Local reconfiguration of aromatic residues triggered upon substrate binding. Substrate binding prompts the closure of EC gates in LeuT (PDB: 2A65), Mhp1 (PDB: 4D1B) and BetP (PDB: 2WIT). In LeuT, isomerization of F253 (*purple*) brings its aromatic side chain into close proximity to Y108 (*green*), closing the thin gate to the EC region. In Mhp1, W117 and W220 rotate towards the substrate, benzylhydantoin (BH). In BetP, four tryptophans sequester the betaine (Bet). *Aromatic* residues in the outward-facing substrate-free state are in *yellow*; those in the substrate-bound form are in *magenta*.

Multiple sequence alignments of TM1 and TM6 (electronic supplementary material, figure S2C) reveal the recurrence of the helix-breaking motif GXG in the TM1 of LeuT (GLG), MhsT (GLG), BetP (GIG), AdiC (GSG) and GadC (GSG), and the TM6 of LeuT (GFG), dDAT (GPGFG), SERT (GPGFG), MhsT (GMG) and ApcT (GFG). The broken regions of these TM helices harbour binding sites for the substrates and ions (figure 2*a–c*). Specific residues (e.g. Glu208 in AdiC [23], Asp79 in hDAT or Asp46 in dDAT [39,40]; figure 1) are required to coordinate the specific substrates, while the backbone carbonyl and amine groups at these irregular regions provide avid sites for binding substrate and/or ions, hence the above-observed sequence specificity across functional families at those helices. Notably, these broken helices are generally composed of small residues Gly, Ser and Ala, instead of the α-helix breaker Pro, which would impart rigidity. The orientational flexibility at the breakage site is essential to enable the transition from OFS to IFS after substrate binding.

Another design principle that apparently further enhances the effectiveness of the broken helices to propagate structural changes induced upon ligand binding is the tight coordination of the bound substrate by bulky/aromatic residues Trp, Phe and Tyr (figures 2*d–f* and 1*c*). These residues reorient to firmly hold the substrate in place to prevent its escape during OFS → IFS reconfiguration. Furthermore, they provide a framework for robustly transmitting the *local* reconfigurations that are triggered upon substrate binding [1,25,27,41] to the arms of the broken helices, thus resulting in the propagation of structural change away from the binding site. A notable example is the tryptophan box in the BCCT family (figure 2*f*) [3].

This analysis thus reveals the three-fold significance of helical disruptions: (i) presenting a high-avidity site for substrate binding originating from the need to satisfy the hydrogen-bond-forming groups; (ii) the high potential to undergo a spatial reorientation change owing to the high flexibility of the GXG motif; and (iii) efficient propagation of structural perturbations to the EC and IC ends by virtue of the tight packing at the hinge centre and rigidity of the two helical arms. Thus, an energetically ‘frustrated’ region which also serves as a hinge-bending centre for reorientation of compact/rigid structural elements (helical arms) on both sides appears as a highly versatile allosteric mechanism for substrate binding-induced reorganization of the tertiary structure. Support for such an allosteric effect is further provided by the change in cross-correlations upon substrate binding. Electronic supplementary material, figure S3 shows how substrate binding induces an increase in the cross-correlation between the movements of the two arms, TM1a and TM1b, of the broken helix TM1.

### (d) Shared fluctuation profile of core residues: a signature of LeuT-fold dynamics

Previous studies have demonstrated that each protein has its own *intrinsic dynamics* uniquely encoded by its overall architecture, or fold, which often facilitates its functional interactions; and the intrinsic dynamics may be analytically evaluated using elastic network models coupled with normal mode analysis [42,43]. Here, we examine the intrinsic dynamics of LeuT-fold structures using the anisotropic network model (ANM) [44].

**Figure 3.**
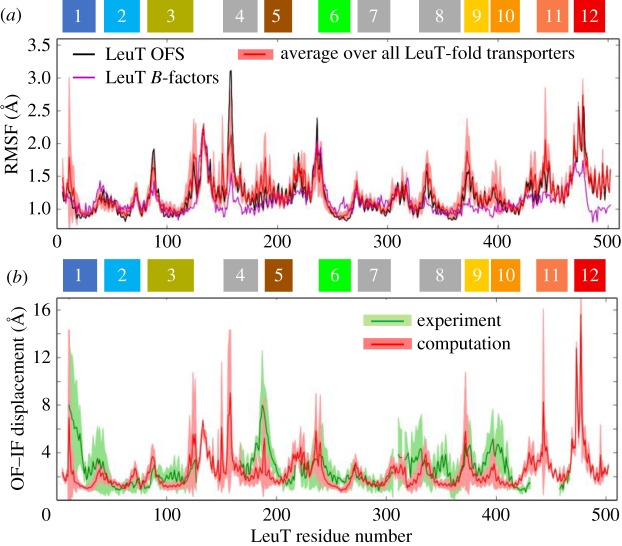
Shared dynamics of LeuT-fold residues from theory and experiments (*a*) RMSF profile for LeuT (*black* line) obtained by ANM analysis of OFS structure (PDB: 2A65, chain A) is compared with the corresponding *B*-factor profile from X-ray crystallography (*purple* line), and the signature profile (*red* line) and its standard deviation (*light red band*) computed for a representative set (electronic supplementary material, table S1). The correlation coefficient between ANM-predicted RMSFs and those derived from *B*-factors is 0.65. (*b*) Comparison of experimental (*green*) and ANM-predicted (*red*) global movements (during OFS ↔ IFS transition) and their variations across LeuT-fold family members. Experimental data refer to LeuT, BetP and Mhp1, resolved in both OFS and IFS; the ANM profile is obtained from the 10 softest modes evaluated for the representative monomers/protomers. Shaded areas indicate the standard deviations.

First, we examined the root-mean-square fluctuation (RMSF) profile of residues for a subset of 11 representative transporters in both OFS and IFS, indicated in electronic supplementary material, table S1. The results are presented in figure 3*a*. The *black* line therein is obtained for LeuT, rescaled based on X-ray crystallographic *B*-factors (*purple* line); and the *red* line (and *light red shading*) represents the average behaviour (and standard deviation) over the entire set. The profiles for the individual transporters can be seen in electronic supplementary material, figure S4. A strong tendency to exhibit the same ‘signature’ profile among all homologues (monomers and protomers) is seen, with small-to-moderate deviations from the mean.

The next question is to what extent this signature profile is used to enable the global transition of the transporters. To address this question, we determined so-called *soft* modes, energetically favoured by the architecture, which often provide paths for cooperative reorganization of the overall structure and enable allosteric effects. A few classical examples of successful representations of allosteric transitions by soft modes predicted by the ANM are the transition of haemoglobin between its T and R states [45], the cooperative conformational changes observed in chaperonin GroEL rings [46] and the opening/closure of adenylate kinase domains [47]; other examples can be found in an earlier review [48], for example. In the present case, structures in both OFS and IFS have been determined for LeuT, BetP and Mhp1, thus allowing quantitative assessment of structural changes involved in the transition OFS ↔IFS (*green* line in figure 3*b*), and comparison with ANM soft modes *(red* line). The comparison reveals that residues’ motions during OFS ↔IFS transition can be traced back to the global modes, or the signature profile, uniquely defined by the LeuT-fold.

A closer inspection shows differences at certain regions, such as TM1, the IC loop between TM4 and TM5, and the EC loop EL4 between TM7 and TM8. The latter participates in regulating EC gating and substrate access [49], a role fulfilled by substrate-specific residues, hence the heterogeneity in the global mode shape at that region. Likewise, the large (approx. 16 Å) displacement of TM1 in the IFS is unique to LeuT in the IFS (electronic supplementary material, figure S4). This movement is much larger than that observed for TM1 in BetP [50], Mhp1 [49] and vSGLT [51]. Structural comparison shows that BetP TM1a is connected to a long helical segment; but in LeuT, it is connected to a disordered tail and therefore enjoys higher mobility. Finally, the TM4–TM5 loop has been observed to unwind/stretch during OFS → IFS transition of LeuT [41], MhsT [30], Mhp1 [52,53] and BetP [50]. The unwound part of TM5 in the conserved motif GlyX_9_Pro of MhsT [30] and an extension of the TM4–TM5 loop (G258-G263) in hDAT [27] have been observed to trigger the hydration of Na2, leading to the opening of the IC vestibule. Such unwinding/disorder at a local scale cannot be reproduced by ANM global modes.

### (e) ANM soft modes provide a complete description of conformational variability observed for LeuT superfamily members

Figure 3*a* demonstrates that LeuT-fold monomers or protomers belonging to different functional families exhibit shared dynamics regardless of their conformational (OFS/IFS) or multimerization (monomer/dimer/trimer) states. Yet, they stabilize the OFS, IFS or intermediate/occluded state and sample a spectrum of conformational changes, during the transport cycle. How are those different conformers compatible with the same fold and signature fluctuation profile?

To gain a mechanistic understanding of the conformational spectrum accessible to LeuT superfamily members, we performed a principal component analysis (PCA) of the ensemble of PDB structures listed in electronic supplementary material, table S1. Optimal superposition of 104 monomers and protomers in this set onto the LeuT OFS structure (PDB: 2A65; *reference* structure) permitted us to identify a core region (electronic supplementary material, figure S1C) and RMSDs from the mean that varied from approximately 1.5 Å for LeuT monomers/protomers to approximately 5 Å for vSGLT, BetP and Mhp1 (electronic supplementary material, figure S5A). Comparison of the results from PCA with ANM predictions (electronic supplementary material, figure S5B) showed that the softest ANM mode (ANM1) computed for the reference structure (the closest to the average structure of the ensemble in terms of RMSD) yields a cumulative overlap of 0.55 with the principal components 2 and 6 (PC2 and PC6).

**Figure 4.**
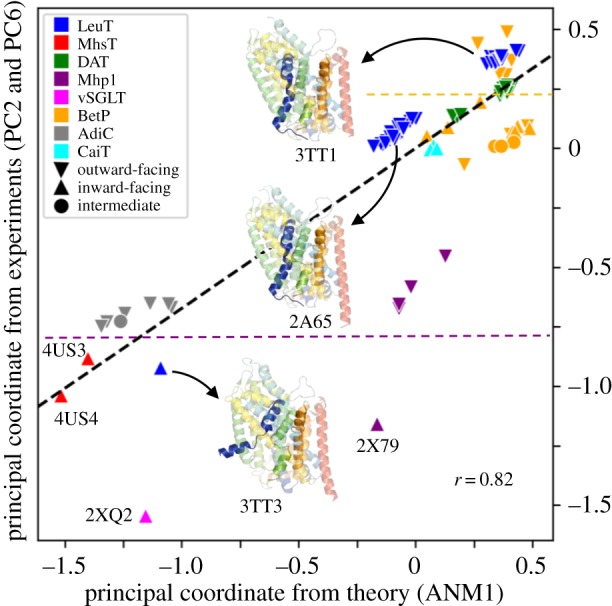
Intrinsic dynamics of the LeuT superfamily explains the structural variability observed experimentally for 104 conformers. Projections of the 104 conformers (electronic supplementary material, Table S1) onto ANM1 and the combined mode from PC2 and PC6 yielded a strong correlation (*r* = 0.82), revealing that the observed differences between these structures comply with the softest mode intrinsically encoded by the LeuT-fold. Colours and shapes represent different families and conformational states, respectively, as described in the legend. Three representative LeuT structures are displayed: outward-facing open (PDB: 3TT1), outward-facing closed (PDB: 2A65) and inward-facing open (PDB: 3TT3), with TM1, 6 and 10 coloured *dark blue*, *orange* and *pink*, respectively.

Figure 4 shows the ensemble of structurally resolved LeuT-fold transporters projected onto the theoretically predicted (ANM1) and experimentally supported (PC2 and PC6 combined) principal modes. The correlation is quite high (0.82), confirming that the two sets describe the same direction of deformation. While members of the same family (see the *colour code*) tend to cluster together, we note that within each family a certain degree of segregation between IF (*upward triangle*) and outward-facing (OF; *downward triangle*) states takes place, for instance in the case of BetP (in *orange*) and Mhp1 (in *purple*), consistent with the analogous separation for LeuT (*blue*). Such observation points to the fact that a common gating mechanism might be shared among members of the superfamily, and is well captured by the softest mode favoured by the common fold.

**Figure 5.**
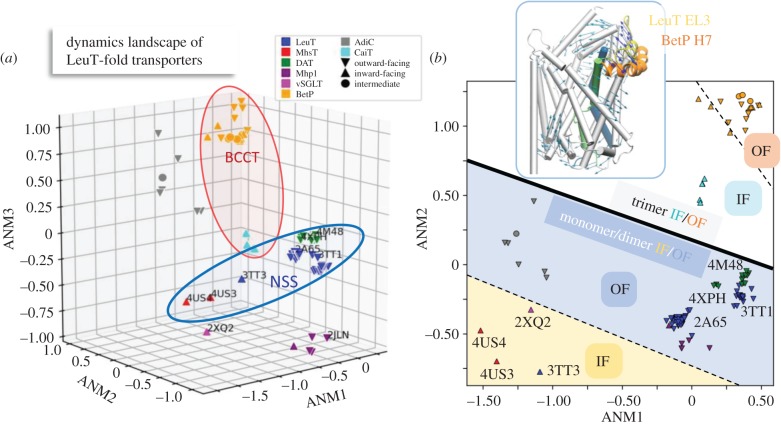
Classification of LeuT-fold transporters based on their collective motions. The distribution of the 104 monomers/protomers are displayed in the subspaces of collective modes spanned by the three (*a*) and two (*b*) softest ANM modes. Panel (*a*) shows the clustering of conformers resolved for the same transporter, or those belonging to the same functional families (enclosed in ellipses). Panel (*b*) provides a clear separation of (i) the monomeric and dimeric transporters (*lower left* portion; *light blue* and *light yellow*) and trimeric transporters (*upper right* portion), and (ii) the inward-facing and outward-facing conformers within each region. ANM mode 2 (see inset) directs the reconfiguration of the LeuT EL3 (loop–helix, *yellow*) along a direction (*blue arrows on the ribbon diagram*) in accord with the structural change undergone by the equivalent BetP H7 helix (*orange*) upon trimerization.

Figure 5*a* provides an overview of the ‘dynamics landscape’ of the LeuT-fold transporters. Therein, all 100+ monomer/protomer structures are projected onto the subspace spanned by ANM1, ANM2 and ANM3, allowing visualization of the different classes of proteins based on their collective motions. Notably, proteins belonging to the same functional family tend to cluster, highlighting the relevance of soft modes to transporter function.

Another interesting fact emerges by focusing on the projection of structures onto the first two ANM modes, shown in figure 5*b*. In this representation, a clear-cut separation can be drawn between trimeric transporters (BetP and CaiT) and monomeric/dimeric transporters, while secondary cuts (dashed lines) further subdivide both groups into OF and IF conformations. This clear separation may be an effect of the structural constraints imposed by the trimeric organization to the monomers of BetP and CaiT transporters, which share a similar quaternary structure (figure 1*c*). The trimers, indeed, feature a different interface compared with dimers, involving the rearrangement of helix H7 in BetP, corresponding to EL3 in LeuT (figure 5*b inset*). Such a rearrangement is well reproduced by ANM2 of LeuT (figure 5*b inset* and electronic supplementary material, figure S6A) as well as ANM3 (electronic supplementary material, figure S6B), suggesting an intrinsic predisposition (via ANM2 and ANM3; see also electronic supplementary material, figure S7) of H7 to adopt the correct positioning for trimeric interface formation.

### (f) Oligomerization facilitates the transition between gating states in LeuT dimer

**Figure 6.**
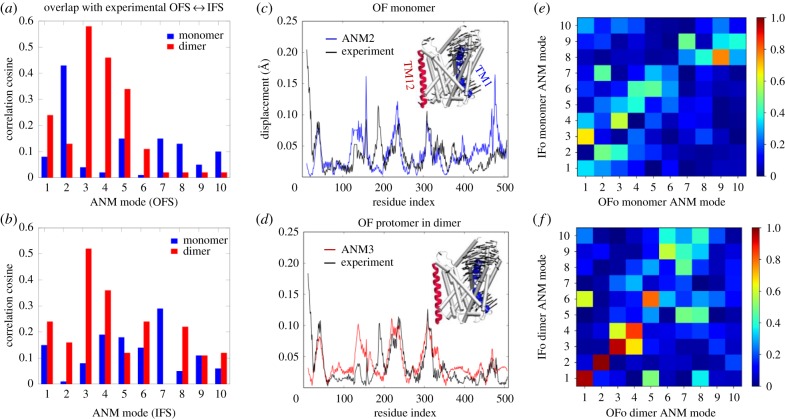
Comparison between ANM modes computed for the monomer and dimer of LeuT. (*a*,*b*) Overlap between ANM modes and respective deformation vectors, computed for the isolated monomer *(blue)* and monomer in the dimer (i.e. protomer, *red*). The conformations in the OFS and IFS are based on the respective PDB structures 3TT1 and 3TT3. (*c*,*d*) Profiles of the (normalized) residue displacements for the deformation vectors (in *black,* also represented by *arrows* on the 3D structures) and for the two ANM modes (in *blue* and *red*) with highest overlap in (*a*). (*e*,*f*) Overlap matrices between OFS and IFS modes evaluated for the monomers (*e*) and protomers in dimers (*f*). Higher overlaps along the diagonal in (*f*) (compared with the diagonal of (*e*)) suggest that the reciprocal interconversion between OFS and IFS is more strongly favoured by the dimeric architecture (compared with monomeric). OFo (IFo), outward (inward) facing open state.

In figure 6*a,b* we compare how well the structural changes observed between OFS and IFS are reproduced by the low-energy modes computed for two models: ANM based on the isolated LeuT monomer (*blue bars*) and ANM for the full dimer (*red bars*). The experimental deformation vectors have been calculated as the difference between the coordinates of LeuT in OFS (PDB: 3TT1, chain A) and IFS (PDB: 3TT3, chain A), after structurally aligning them in both monomeric and dimeric form. This alignment procedure allows a direct comparison of the respective monomeric/dimeric ANMs.

The *bar plots* in figure 6*a*,*b* clearly show that the dimeric configuration boosts the displacement propensity of the protomer along the direction of the deformation observed in crystal structures. ANM3 in the dimer, in particular, yields an overlap (cosine correlation) as high as approximately 0.6 (in *red*), compared with a maximum overlap of approximately 0.4 for ANM2 of the isolated monomer (in *blue*). This result is even more notable after considering that ANM3 in the dimer is the first genuine mode describing internal conformational changes of the protomer, because modes 1 and 2 basically involve quasi-rigid rotations of the two protomers around a central axis at the dimer interface, illustrated in electronic supplementary material, figure S8.

Figure 6*c*,*d* clarify that the main effect of dimerization is to suppress the mobility of the helices involved in the dimer interface (especially TM12, in *red* in the 3D structure), in agreement with what is observed in the crystal structures. Interestingly, neither monomeric nor dimeric main ANM modes could reproduce the opening of TM1 (in *blue* on the structure), corresponding to the highest peak in the far-left portion of the deformation vectors profile. Such large displacements of TM1, however, are reproduced by using the IF conformation of LeuT, as explained above.

Figure 6*e*,*f* reveal a better reciprocal overlap between the two sets of soft modes accessible to OFS and IFS in the case of the dimeric model, compared with the monomeric one, suggesting that a reversible interconversion between the two gating states is favoured in the dimer.

## 3. Conclusion

The present study focused on a superfamily of structural homologues, LeuT superfamily, that encompasses members from five families of transporters with different functions and low sequence identity. We first characterized their shared structural and dynamic characteristics, and then proceeded to elucidate which features on a local or global scale, structural or dynamic, differentiate them to lead to different functions. The first task helped us identify a signature residue–fluctuation profile (figure 3) intrinsically favoured by their common fold, consistent with the postulate that shared fold also implies shared global dynamics. This also implies that those transporters or antiporters have evolved to recruit the same tertiary fold, despite their sequence dissimilarities, presumably driven by the adaptability of the fold to chemical (specificity) and physical (conformational flexibility) differences, thus allowing functional differentiation.

Chemical specificity can be detected at the dissimilar sequence patterns among superfamily members that belong to different functional families, while those transporters within a given family exhibit distinctively higher sequence identities. The difference becomes even more pronounced upon focusing on TM helices involved in substrate/ion binding: similarities among the same functional family members are enhanced, while dissimilarities across different functional families become even more pronounced (electronic supplementary material, figure S2).

Physical flexibility, on the other hand, is manifested by structural differences between family members on both a global (OFS/IFS and intermediate states; multiple oligomerization states; figure 1 and electronic supplementary material, figure S1) and a local (helical disruptions, substrate coordination geometry; figure 2) scale. It is only upon substrate/ligand binding that the pre-existing signature fluctuations are advantageously exploited to drive the transport of substrate. First, EC gate closure is triggered, and then further insertion of the ion/substrate binding to a structurally irregular, broken helical, region confers a local reordering (e.g. TM1 tilting, or TM6 reorientation) that propagates to the IC region upon the rigidification of the originally frustrated cluster of residues; and the induced structural change opens the IC gate to trigger an influx of IC water, which stimulates the removal of substrate/ion and its release to the IC region. Thus a cascade of events takes place, stimulated upon substrate and ion binding, typical of the cooperative response of allosteric proteins to ligand binding.

A rigorous examination of the distribution of LeuT superfamily members in the conformational space accessible to them (figures 4 and 5) demonstrates how the resolved structures are essentially reorganizations of the shared fold along the softest ANM modes 1, 2 and 3 intrinsically favoured by their shared fold. ANM1 provides a good description of the principal variations in structure elucidated by the PCA of 104 monomers/protomers (figure 4); ANM2 plays a dominant role in distinguishing the trimeric transporters (figure 5*b*); and ANM3 together with ANM2 helps the transition between OFS and IFS, as evidenced by electronic supplementary material, figure S6. This analysis shows that the adaptation of the shared fold to different conformational or oligomerization states is mainly accomplished by the soft paths of reconfiguration intrinsically encoded by the LeuT-fold.

On a broader scale, this study provides an example of adaptability of structures to different functions by virtue of their intrinsic flexibility, as recently reviewed [54], and pointed out to be the case between the AMPAR and NMDAR families of ionotropic glutamate receptors [55]. Suitable substitutions of residues combined with structural malleability help accomplish the biological function in alternative ways. For example, LeuT-fold members also function as Na^+^-independent antiporters or H^+^-coupled symporters. Specific amino acids apparently serve the same functional roles as the *co*-transported ions, suggesting common principles among ion-coupled or -uncoupled transporters [1,3,56], e.g. replacement of Glu by Ser enables Cl^−^-dependent activity in a LeuT mutant (E290S) [57]; a methionine sulfur in CaiT (Met331) [3] and ApcT [56] consistently occupies the Na1 site, suggesting that it replaces the Na1 [3]; the Na2 position in Na^+^-independent CaiT and ApcT is occupied by the positively charged Arg262 in CaiT [3] or Lys158 in ApcT [56].

The dynamics landscape generated here for the ensemble of LeuT-fold transporters (figure 5) allows a classification of transporters based on their collective dynamics. Notably, transporters belonging to the same functional family tend to cluster in accord with the relevance of soft modes to function. The landscape further provides a clear view of the soft modes involved in functional changes or oligomerization. We note, however, that ANM analysis is not suitable for (i) very small proteins or peptides where chemical specificity becomes important, (ii) large-scale domain movements that involve a passage over relatively high-energy barriers beyond those surmounted by coarse-graining, or (iii) systems whose dynamics is significantly perturbed by environmental effects, e.g. constraints exerted by the lipid bilayer on intrinsic (lateral) movements of membrane proteins, as noted in glutamate transporter Glt_Ph_ [58]. The need to take account of the lipid, or the environment in general, has been addressed in a recent extension of ANM implemented in the DynOmics server [59].

Oligomerization of NSSs has been suggested to be a determinant of transporter trafficking to the plasma membrane in addition to enabling efficient substrate transport [60,61]. While the resolved dDAT [9] or hSERT [62] structures are monomeric, growing data (including radiation inactivation, cross-linking, mutagenesis, co-immunoprecipitation; see review [60]) and single-molecule experiments [63]) suggest that NSS family members may exist and function as oligomers. Likewise, even though the monomeric BetP is active, BetP requires the trimeric form to properly respond to osmotic stress, indicating the role of trimerization for transport regulation [64]. Our study sheds light on the intrinsic ability of LeuT superfamily members to form multimers (e.g. the reconfiguration of the periplasmic α-helix 7 (H7, which mediates trimerization via ANM3 in figure 5 and electronic supplementary material, figure S6), and further suggests that oligomerization may allosterically alter or enhance functional changes in structure, shown here for the dimerization of LeuT (figure 6). Finally, lipid binding-mediated oligomerization of membrane proteins is critical in many cell-signalling pathways [65]. There is compelling need to further investigate the functional significance of oligomeric states and binding of accessory substrates or lipids that may further modulate their allosteric cooperativity among the protomers.

## 4. Methods

We used several modules in ProDy [66] for performing various tasks, including structural alignments, PCA and ANM analyses, and comparisons with experimental deformations. Details are provided in the electronic supplementary material. All the software used here are accessible online.

## Supplementary Material

Supplementary Material

## Data accessibility

This article has no additional data.
