# Supplementary material for: Shared dynamics of LeuT superfamily members and allosteric differentiation by structural irregularities and multimerization
Source: Philos Trans R Soc Lond B Biol Sci. 2018 May 7;373(1749):20170177. doi: 10.1098/rstb.2017.0177 (PMC5941172; doi:10.1098/rstb.2017.0177)
Supplement: Supplementary Material [file rstb20170177supp1.docx]

**Supplemental Material**

**Shared Dynamics of LeuT Superfamily Members and Allosteric Differentiation by Structural Irregularities and Multimerization**

**Luca Ponzoni,* She Zhang,* Mary H. Cheng,* Ivet Bahar ^#^**

*Department of Computational and Systems Biology, School of Medicine, University of Pittsburgh, Pittsburgh, PA 15213*

^*^ equal contribution

^#^ Corresponding author

Dr. Ivet Bahar

Distinguished Professor and John K. Vries Chair

Computational & Systems Biology

School of Medicine, University of Pittsburgh

3064 Biomedical Science Tower 3

3501 Fifth Avenue, Pittsburgh, PA 15213

Voice: 4126483332 - Fax: 4126483163

[bahar@pitt.edu](mailto:bahar@pitt.edu); http://www.ccbb.pitt.edu/Faculty/bahar/

**Supplemental Method**

***Sequence alignment.*** The ten TM helices (TM1-TM10) shared by the LeuT-fold family are designated following the convention adopted for numbering LeuT helices [1]. For example, TM3 to TM7 and TM8 to TM12 in CaiT or BetP are the equivalent TM1 to TM5, and TM6 to TM10 of the inverted repeat in the family. Multiple sequence alignments for the transporters (**Fig. S2**), or for the selected TM1 (**Table S2**) or TM6 (**Table S3**) helices were performed using Clustal Omega [2].

***RMSF profile.*** For the analysis of the shared dynamics presented in **Figs 3** and **S4**, 11 representative transporters indicated in boldface in **Table S1** have been structurally aligned using the STAMP algorithm [3] from the MultiSeq tool [4] of VMD [5]. The ANM modes have been then computed on each single structure, using the ProDy Python application programming interface (API) [6, 7]. In a few transporters, some short sequence segments at the termini have been excluded, to avoid interference with the ability of ANM analysis to capture global motions (e.g., in BetP the protruding helix formed by residues 544-583 has not been included in the network model). ANM yields the distribution of RMSFs, but not their absolute size. The latter is adjusted by rescaling the uniform force constant γ with respect to B-factors, which led to γ/kT = 0.13/Å^2^ in **Fig 3A**. The average profiles in **Fig 3B** for the theoretical RMSFs (*red* *curve*) and the experimental displacements between OFS and IFS (*green* *curve*) have been obtained by combining individual profiles (11 and 3 curves, respectively) at each sequence site, whenever at least two residues were available that could be mapped to a LeuT residue.

***Construction and Principal Component Analysis of the ensemble of conformers in* Figs 4-5**. From each transporter family (LeuT, DAT, MhsT, Mhp1, vSGLT, BetP, CaiT, AdiC), we first choose the structure with the most complete sequence as a representative to be structurally aligned against the reference structure [8] (PDB:2A65; an outward-facing occluded LeuT). We used the CE algorithm [9], which is integrated into PyMol (https://pymol.org/), to perform the structure alignment. The other PDB structures were then sequentially aligned with respect to the family representative. BetP structures are all trimers, and they are often asymmetric; therefore, each protomer was included separately in the ensemble. For the other oligomeric structures, only one protomer was included. This led to an ensemble of 104 conformers (of monomers or protomers). We obtained a multiple sequence alignment of these conformers that permitted to characterize the structural variance of each conformer. The structural alignment was iteratively improved to minimize the RMSD values of individual conformers, using the ProDy API [6, 7]. Principal Component Analysis (PCA) of the ensemble of conformers was performed by evaluating and eigenvalue decomposing the 3*N* x 3*N* covariance matrix generated for the ensemble, where *N* = 412, using the ProDy API.

***ANM analysis of the ensemble of conformers***. The lowest frequency modes based on the anisotropic network model (ANM) [10] were calculated using ProDy [6, 7] and visualized using Normal Mode Wizard module implemented in VMD [5]. The ANM Hessian matrix was built using all C^α^ atoms and a pairwise interaction cutoff of 15 Å. For ANM analysis, the reference structure was trimmed using the reduced model [11] implemented in ProDy to ensure proper matching of residues between the conformers and the reference structure, while maintaining the accuracy of the predicted dynamics upon considering the trimmed residues as environment.

***ANM analysis of LeuT monomer and dimer.*** The deformation vector describing the structural changes between the OFS and IFS of LeuT monomer (**Fig 6C**) has been computed after optimally aligning the two LeuT monomeric structures in the PDB structures 3TT1 and 3TT3, respectively: ***v***_def_^mon^ = ***x*** ^OF^ – ***x*** ^IF^. Analogously, in order to compute the deformation vector for the protomer in the dimer (**Fig 6D**), the same two LeuT structures have been aligned in dimeric form: ***v***_def_^dim^ = ***x****’* ^OF^ – ***x****’* ^IF^. For the calculation of the overlap *O* between the deformation vectors and a generic ANM mode ***v***_ANM_ (**Figs 6A-B**), or that between ANM modes (**Figs 6E-F**), the cosine correlation (dot product) has been computed between the two normalized vectors, discarding potential minus sign arising from the arbitrary choice of sign for ANM modes: *O =* |***v***_def_ ∙ ***v***_ANM_ |. In the dimer case, both vectors have been sliced to include coordinates of only one protomer.

**Supplemental Tables**

**Table S1. Ensemble of LeuT-fold transporters examined in the present study, and corresponding functional families and oligomerization and conformational states**

| **Oligomeric state** | **Family** | **Transporter** | **Conformation ^(a)^** | **PDB structures ^(b)^** |
| --- | --- | --- | --- | --- |
| **trimer** | BCCT | BetP^[12-14]^ | OF*o* | 4LLH |
|  |  |  | intermediate | 2WIT |
|  |  |  | asym (IF*o*/IF*o*/OF*o*) | 3P03 |
|  |  |  | asym (IF*c*/OF/IF*o*) | 4AIN |
|  |  |  | asym (IF/IF/OF) | **4C7R** |
|  |  |  | asym (OF*c*/OF*o*/IF*o*) | **4DOJ** |
|  |  | CaiT^[1, 15]^ | IF*o* (subs. bound) | 3HFX |
|  |  |  | IF*o* | 4M8J, **2WSX**, 2WSW |
| **dimer** | APC | AdiC^[16, 17]^ | OF*c* (subs. bound) | 3L1L |
|  |  |  | OF | 3NCY, 3LRB, 3LRC, **5J4I**, 5J4N |
|  |  |  | intermediate | 3OB6 |
|  | SSS | vSGLT^[18, 19]^ | IF*po* | **2XQ2** |
|  | NSS | LeuT^[8, 20-22]^ | OF*c* (subs. bound) | **2A65**, 2Q6H, 2Q72, 2QB4, 2QEI, 2QJU, 3F3C, 3F3D, 3F3E, 3F48, 3F4I, 3F4J, 3GJC, 3GJD, 3GWU, GWV, GWW, 3MPN, 3MPQ, 3QS5, 3QS6, 3TU0, 3USG, 3USI, 3USJ, 3USK, 3USL, 3USM, 3USO, 3USP, 4HMK, 4HOD |
|  |  |  | OF*o* | 3TT1, 3F3A, 4MM4, 4MM5, 4MM6, 4MM7, 4MM8, 4MM9, 4MMA, 4MMB, 4MMC, 4MMD, 4MME, 4MMF |
|  |  |  | OF | 4FXZ, 4FY0, 3QS4 |
|  |  |  | IF | **3TT3**^(c)^ |
| **monomer** | NSS | DAT^[23, 24]^ | OF | 4M48, 4XNU, 4XNX, 4XP1, 4XP4, 4XP5, 4XP6, **4XP9**, 4XPA, 4XPB, 4XPF, 4XPG, 4XPH, 4XPT |
|  |  | MhsT^[25]^ | IF | 4US3, **4US4** |
|  | NCS1 | Mhp1^[26, 27]^ | OF | 2JLN, **4D1A**, 4D1B, 4D1C, 4D1D |
|  |  |  | IF | **2X79** |

*^(a)^ OF: outward facing; IF: inward facing; suffixes o and c refer to the open or closed states of the EC (in the OF state) or IC (in the IF state) gates; po is partially open. Structures resolved in the presence of substrate are indicated; ^(b)^* *PDB codes. Those used in structural alignments (Fig S1) and in the generation of the fluctuation profiles (Figs 3 and S4), referred to as representative 11 structures (monomers or protomers), are indicated in boldface. LeuT may presumably function in either monomeric or dimeric state, but the majority of the resolved structures are dimeric. ^(c)^ LeuT IF dimer [28, 29] computed from IF monomer 3TT3. Overall the Table contains 104 distinctive conformers belonging to monomers or multimers, which have been used in generating Figs 4, 5, and S7).*

**Table S2: Percent Identity Matrix for TM1 helices of LeuT superfamily members**

|  | LeuT | dDAT | hSERT | MhsT | ApcT | AdiC | BetP | CaiT | Mhp1 | vSGLT |
| --- | --- | --- | --- | --- | --- | --- | --- | --- | --- | --- |
| LeuT | 100 | 36 | 40 | 60 | 14.29 | 28 | 16.67 | 8.33 | 18.18 | 18.75 |
| dDAT | 36 | 100 | 84 | 44 | 4.76 | 20 | 4.17 | 4.17 | 27.27 | 12.5 |
| hSERT | 40 | 84 | 100 | 48 | 4.76 | 16 | 8.33 | 4.17 | 22.73 | 12.5 |
| MhsT | 60 | 44 | 48 | 100 | 19.05 | 16 | 12.5 | 12.5 | 27.27 | 25 |
| ApcT | 14.29 | 4.76 | 4.76 | 19.05 | 100 | 23.81 | 14.29 | 14.29 | 11.11 | 25 |
| AdiC | 28 | 20 | 16 | 16 | 23.81 | 100 | 16.67 | 8.33 | 18.18 | 6.25 |
| BetP | 16.67 | 4.17 | 8.33 | 12.5 | 14.29 | 16.67 | 100 | 44 | 9.09 | 18.75 |
| CaiT | 8.33 | 4.17 | 4.17 | 12.5 | 14.29 | 8.33 | 44 | 100 | 4.55 | 18.75 |
| MhP1 | 18.18 | 27.27 | 22.73 | 27.27 | 11.11 | 18.18 | 9.09 | 4.55 | 100 | 33.33 |
| vSGLT | 18.75 | 12.5 | 12.5 | 25 | 25 | 6.25 | 18.75 | 18.75 | 33.33 | 100 |

**Table S3: Percent Identity Matrix for TM6 helices of LeuT superfamily members**

|  | LeuT | dDAT | hSERT | MhsT | ApcT | AdiC | BetP | CaiT | Mhp1 | vSGLT |
| --- | --- | --- | --- | --- | --- | --- | --- | --- | --- | --- |
| LeuT | 100 | 48 | 56 | 68 | 28 | 50 | 19.05 | 4.76 | 46.15 | 33.33 |
| dDAT | 48 | 100 | 80 | 40 | 28 | 20 | 19.05 | 4.76 | 7.69 | 16.67 |
| hSERT | 56 | 80 | 100 | 40 | 32 | 40 | 23.81 | 4.76 | 30.77 | 22.22 |
| MhsT | 68 | 40 | 40 | 100 | 20 | 40 | 14.29 | 4.76 | 15.38 | 22.22 |
| ApcT | 28 | 28 | 32 | 20 | 100 | 20 | 23.81 | 9.52 | 7.69 | 16.67 |
| AdiC | 50 | 20 | 40 | 40 | 20 | 100 | 15.38 | 15.38 | 19.05 | 21.43 |
| BetP | 19.05 | 19.05 | 23.81 | 14.29 | 23.81 | 15.38 | 100 | 40 | 12.5 | 10.53 |
| CaiT | 4.76 | 4.76 | 4.76 | 4.76 | 9.52 | 15.38 | 40 | 100 | 18.75 | 10.53 |
| Mhp1 | 46.15 | 7.69 | 30.77 | 15.38 | 7.69 | 19.05 | 12.5 | 18.75 | 100 | 30.77 |
| vSGLT | 33.33 | 16.67 | 22.22 | 22.22 | 16.67 | 21.43 | 10.53 | 10.53 | 30.77 | 100 |

**Supplemental Figures**


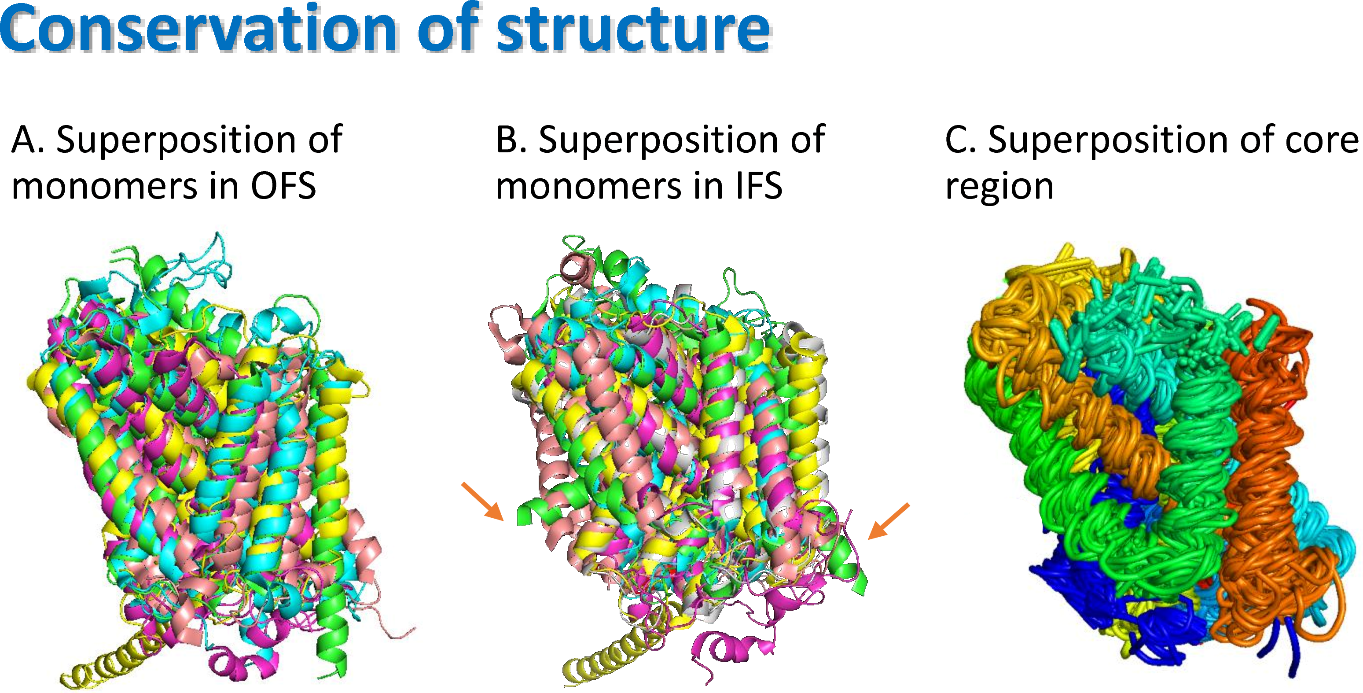


**Figure S1. Structural alignment of LeuT-fold transporters in the OFS (A) or IFS (B), and superposition of their core region (C). (A)** Superposition of LeuT (*green*), DAT (*indigo*), Mhp1 (*magenta*), BetP (*yellow*) and AdiC (*pink*) monomer/protomers in the OFS. (**B**) Superposition of LeuT (*green*), MhsT (*indigo*), Mhp1 (*magenta*), BetP (*yellow*), vSGLT (*pink*), CaiT (*white*) monomers/protomers resolved in the IFS. (**C**) Core residues selected based on structural alignment of all structures listed in boldface in **Table S1**, and adopted for evaluating conserved dynamics.

**Figure S2. Sequence comparison of LeuT superfamily transporters.** Panel (**A**) displays the pairwise sequence identities (scale bar on the *right*) for all transporters listed in **Table S1**, plus hSERT and ApcT also sharing the LeuT fold, and belonging to the respective families of NSS and APC. The corresponding percent identities are shown on the *lower* matrix (panel **(B)**) Those pairs exhibiting sequence identities above 20% are colored. Panel (**C**) displays the alignment of two TM helices, TM1 and TM6, critical for substrate binding, specificity and translocation. Residues at the helical disruption regions (mostly containing GXG motifs) are highlighted in *green*; and TM6 aromatic residues involved in substrate stabilization are colored *violet*. Panels **(D)-(E)** display the corresponding sequence identities. See also **Fig 2** and **Table S2-S3**.


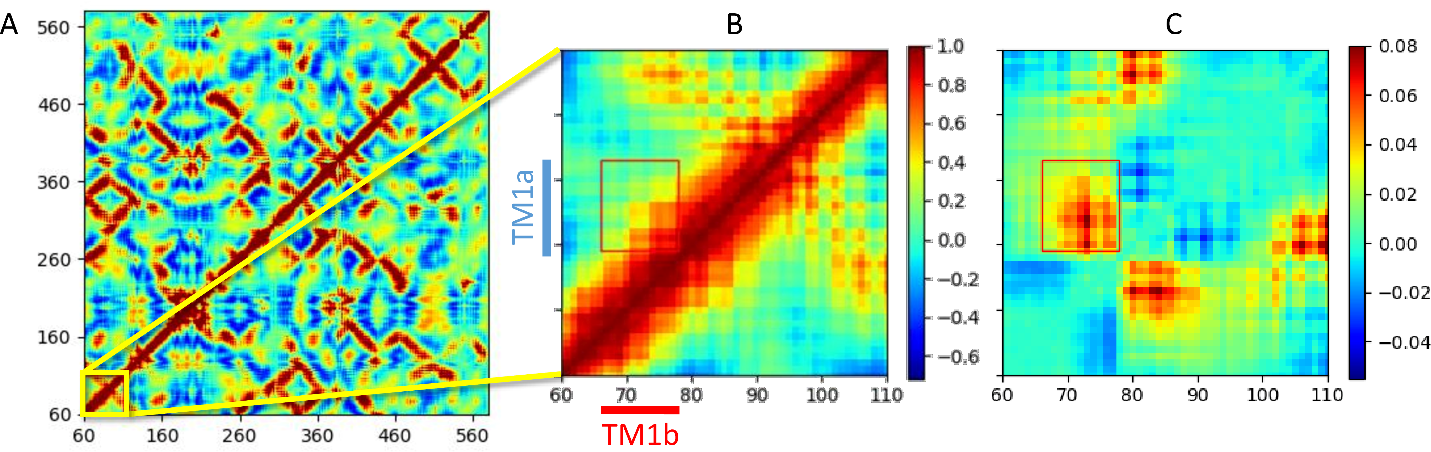


**Figure S3.** **Enhancement of cross-correlations between the motions of the two arms TM1a and TM1b of the broken helix TM1 in DAT upon substrate binding.** **(A)** Covariance matrix between residue movements obtained by the ANM for DAT in the absence of substrate (dopamine). **(B)** Close-up view of the cross-correlations around broken helix TM1, again in the absence of substrate. TM1 is formed by the two segments, TM1a and TM1b, whose residue ranges are indicated, and the corresponding region of the cross-correlation map are indicated by the red box. **(C)** Change in the cross-correlations induced upon substrate binding. The map represents the difference between the substrate-bound and unbound DAT dynamics. Upon substrate binding, increased cross-correlations are observed between segments TM1a and TM1b, as indicated by the red spots in the highlighted region. The substrates (and 2 Na^+^ ions, and one Cl^-^ ion) have been modelled by adding 6 nodes to the ANM at the substrate/ion binding site.


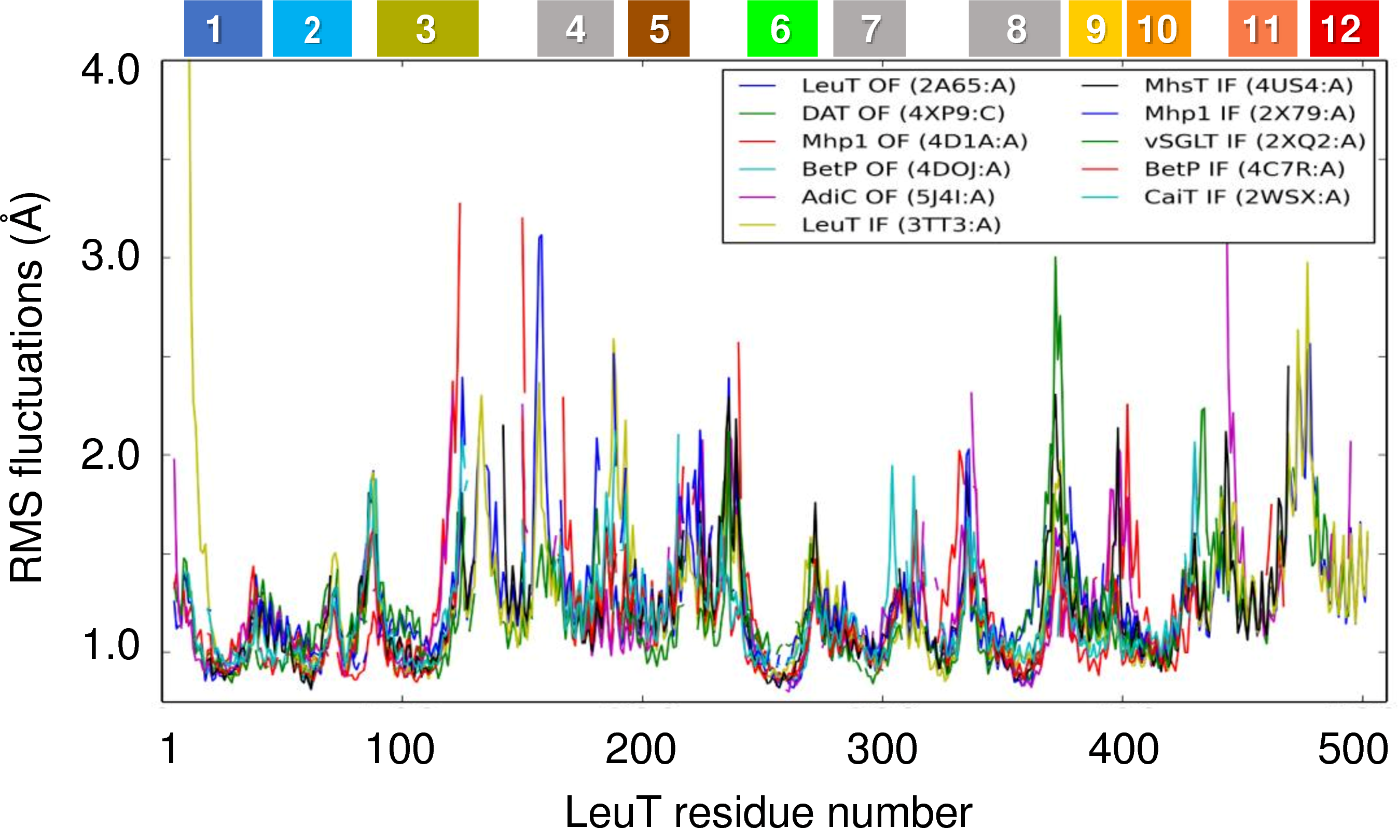


**Figure S4. Comparison of RMSFs generated for representative members of LeuT-fold superfamily.** RMSF profiles for 11 structures representing the 8 transporters with LeuT fold, resolved in both OFS and IFS (when available), were evaluated using the ANM. The inset lists the representative structures, their PDB identities, and selected chains. Their average signature profile derived from these curves is presented in **Fig 3A** (*red* line).


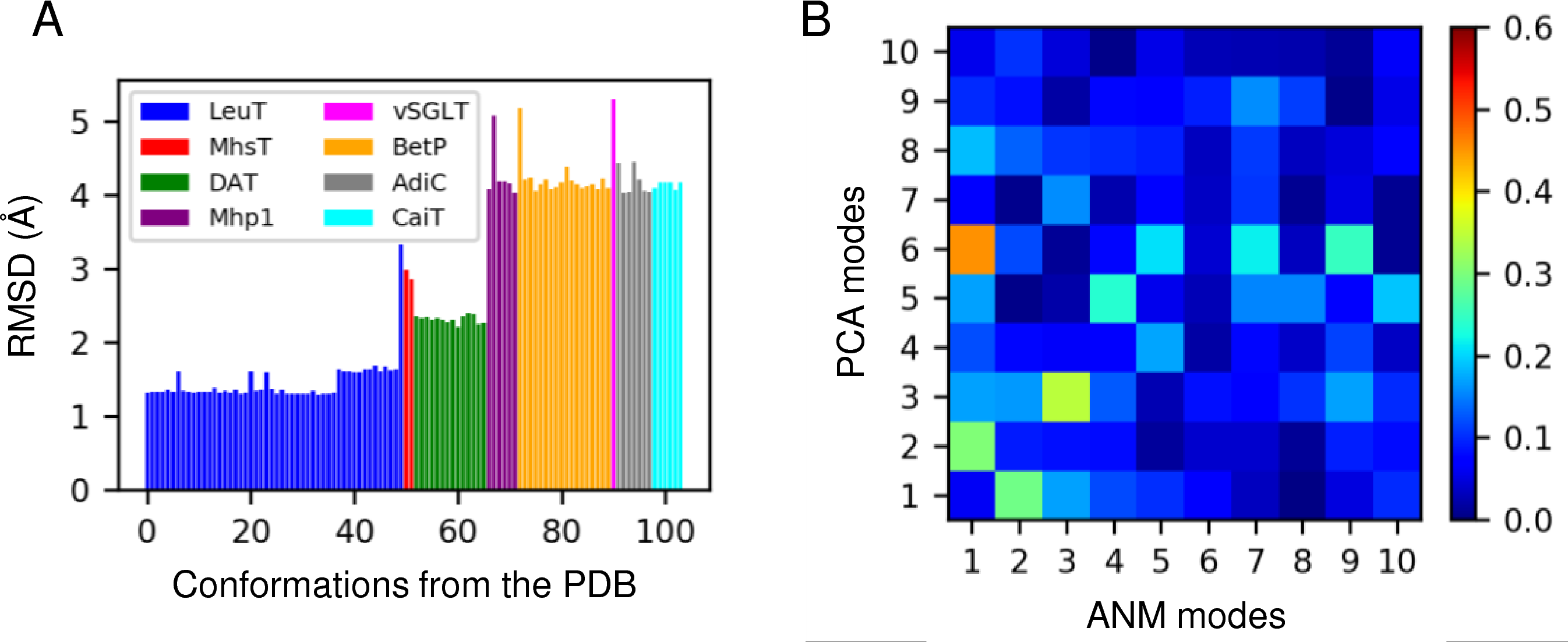


**Figure S5. Structural variations among LeuT-fold family members and intrinsically favored modes of motion.** **(A)**RMSDs calculated for all monomers and protomers in the structural ensemble with respect to the average. The heterogeneous structural ensemble was built by structurally aligning 104 structures with respect to the reference (PDB ID: 2A65). Sequence alignment alone was not sufficient, due to low sequence similarities among different families (see **Fig S2A**). **(B)**Overlaps between ANM and PCA modes. ANM modes were calculated for the reference structure. PCA modes were deduced from the ensemble of 104 monomers/protomers in experimentally resolved structures, listed in **Table S1**.


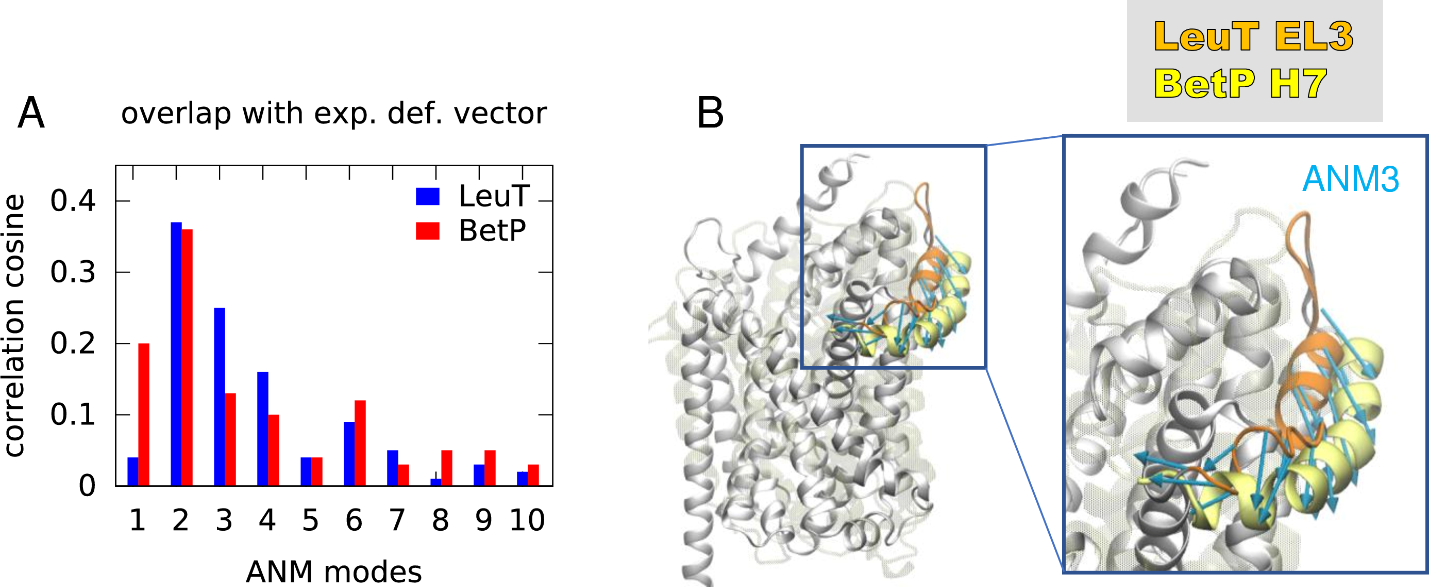


**Figure S6.**  **The role of intrinsic modes ANM2 and ANM3 in enabling the reconfiguration of BetP H7 for making interfacial contacts in the trimeric BetP.** (**A**) Overlap between the structural variation between the experimental structures of LeuT and BetP, and their respective ANM modes. (**B**) LeuT intrinsic mode 3 (ANM3) overlaps with the deformation which characterizes the structural difference between LeuT EL3 and BetP H7, as also observed in **Fig 5** for ANM mode 2 and as quantified in panel **A**.


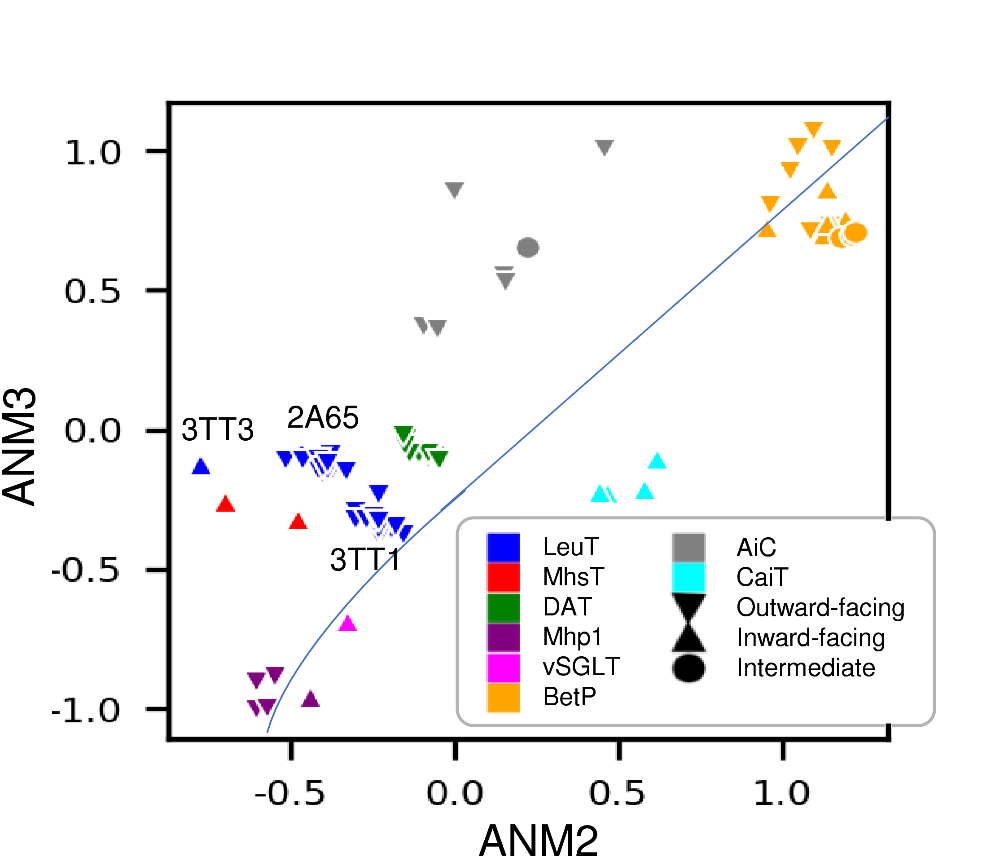


**Figure S7.**  **Projection of LeuT superfamily structures onto the subspace spanned by ANM modes 2 and 3**. The *blue* line separates the conformational space into two regions populated by transporters in the IFS (*triangular*, *lower right*) and OFS (*inverse triangular; upper left*). The exceptions in the latter region are the IF structures of LeuT (3TT3) and the two structures resolved for MhsT in the IFS (*red triangles*). The latter are distinguished from structures in the OFS conformers by their distinctive positions along the ANM1 (see **Fig 4**).


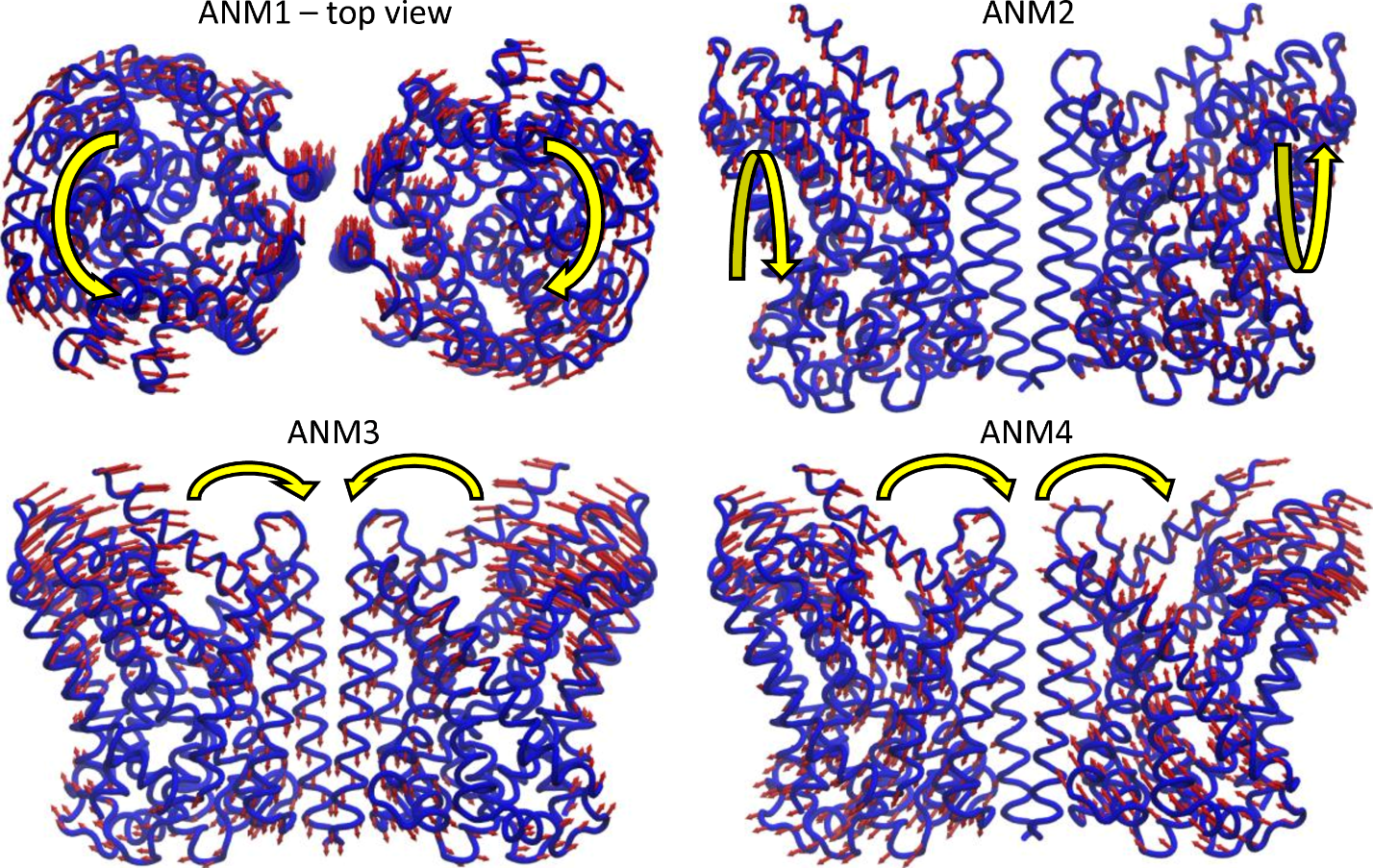


**Figure S8. Schematic representation of the collective motions driven by the first 4 ANM modes of LeuT dimer.** The first two modes correspond to rigid rotations of the two monomers around a central axis at the dimer interface. Instead, both modes 3 and 4 describe a similar “internal” motion of each monomer, the opening of the outward-facing side, in an anti-correlated and correlated fashion, respectively.

**References**

[1] Schulze, S., Koster, S., Geldmacher, U., Terwisscha van Scheltinga, A. C. & Kuhlbrandt, W. 2010 Structural basis of Na+-independent and cooperative substrate/product antiport in CaiT. *Nature* **467**, 233-236.

[2] Sievers, F., Wilm, A., Dineen, D., Gibson, T. J., Karplus, K., Li, W., Lopez, R., McWilliam, H., Remmert, M. & Söding, J. 2011 Fast, scalable generation of high‐quality protein multiple sequence alignments using Clustal Omega. *Mol. Syst. Biol.* **7**, 539.

[3] Russell, R. B. & Barton, G. J. 1992 Multiple protein sequence alignment from tertiary structure comparison: assignment of global and residue confidence levels. *Proteins: Structure, Function, and Bioinformatics* **14**, 309-323.

[4] Roberts, E., Eargle, J., Wright, D. & Luthey-Schulten, Z. 2006 MultiSeq: unifying sequence and structure data for evolutionary analysis. *BMC bioinformatics* **7**, 382.

[5] Humphrey, W., Dalke, A. & Schulten, K. 1996 VMD: Visual molecular dynamics. *J. Mol. Graph.* **14**, 33-38.

[6] Bakan, A., Meireles, L. M. & Bahar, I. 2011 ProDy: protein dynamics inferred from theory and experiments. *Bioinformatics* **27**, 1575-1577.

[7] Bakan, A., Dutta, A., Mao, W., Liu, Y., Chennubhotla, C., Lezon, T. R. & Bahar, I. 2014 Evol and ProDy for bridging protein sequence evolution and structural dynamics. *Bioinformatics* **30**, 2681-2683.

[8] Yamashita, A., Singh, S. K., Kawate, T., Jin, Y. & Gouaux, E. 2005 Crystal structure of a bacterial homologue of Na+/Cl--dependent neurotransmitter transporters. *Nature* **437**, 215-223.

[9] Shindyalov, I. N. & Bourne, P. E. 1998 Protein structure alignment by incremental combinatorial extension (CE) of the optimal path. *Protein Eng.* **11**, 739-747.

[10] Atilgan, A. R., Durell, S. R., Jernigan, R. L., Demirel, M. C., Keskin, O. & Bahar, I. 2001 Anisotropy of fluctuation dynamics of proteins with an elastic network model. *Biophys. J.* **80**, 505-515.

[11] Zheng, W. & Brooks, B. R. 2005 Probing the local dynamics of nucleotide-binding pocket coupled to the global dynamics: myosin versus kinesin. *Biophys. J.* **89**, 167-178.

[12] Ressl, S., Terwisscha van Scheltinga, A. C., Vonrhein, C., Ott, V. & Ziegler, C. 2009 Molecular basis of transport and regulation in the Na+/betaine symporter BetP. *Nature* **458**, 47-52.

[13] Perez, C., Koshy, C., Yildiz, O. & Ziegler, C. 2012 Alternating-access mechanism in conformationally asymmetric trimers of the betaine transporter BetP. *Nature* **490**, 126-130.

[14] Koshy, C., Schweikhard, E. S., Gärtner, R. M., Perez, C., Yildiz, Ö. & Ziegler, C. 2013 Structural evidence for functional lipid interactions in the betaine transporter BetP. *EMBO J.* **32**, 3096-3105.

[15] Kalayil, S., Schulze, S. & Kühlbrandt, W. 2013 Arginine oscillation explains Na+ independence in the substrate/product antiporter CaiT. *Proc. Natl. Acad. Sci. U.S.A.* **110**, 17296-17301.

[16] Gao, X., Lu, F., Zhou, L., Dang, S., Sun, L., Li, X., Wang, J. & Shi, Y. 2009 Structure and Mechanism of an Amino Acid Antiporter. *Science* **324**, 1565-1568.

[17] Gao, X., Zhou, L., Jiao, X., Lu, F., Yan, C., Zeng, X., Wang, J. & Shi, Y. 2010 Mechanism of substrate recognition and transport by an amino acid antiporter. *Nature* **463**, 828-832.

[18] Faham, S., Watanabe, A., Besserer, G. M., Cascio, D., Specht, A., Hirayama, B. A., Wright, E. M. & Abramson, J. 2008 The Crystal Structure of a Sodium Galactose Transporter Reveals Mechanistic Insights into Na<sup>+</sup>/Sugar Symport. *Science* **321**, 810-814.

[19] Watanabe, A., Choe, S., Chaptal, V., Rosenberg, J. M., Wright, E. M., Grabe, M. & Abramson, J. 2010 The mechanism of sodium and substrate release from the binding pocket of vSGLT. *Nature* **468**, 988-991.

[20] Krishnamurthy, H. & Gouaux, E. 2012 X-ray structures of LeuT in substrate-free outward-open and apo inward-open states. *Nature* **481**, 469-474.

[21] Singh, S. K., Yamashita, A. & Gouaux, E. 2007 Antidepressant binding site in a bacterial homologue of neurotransmitter transporters. *Nature* **448**, 952-956.

[22] Singh, S. K., Piscitelli, C. L., Yamashita, A. & Gouaux, E. 2008 A Competitive Inhibitor Traps LeuT in an Open-to-Out Conformation. *Science* **322**, 1655-1661.

[23] Penmatsa, A., Wang, K. H. & Gouaux, E. 2013 X-ray structure of dopamine transporter elucidates antidepressant mechanism. *Nature* **503**, 85-90.

[24] Wang, K. H., Penmatsa, A. & Gouaux, E. 2015 Neurotransmitter and psychostimulant recognition by the dopamine transporter. *Nature* **521**, 322-327.

[25] Malinauskaite, L., Quick, M., Reinhard, L., Lyons, J. A., Yano, H., Javitch, J. A. & Nissen, P. 2014 A mechanism for intracellular release of Na+ by neurotransmitter/sodium symporters. *Nat. Struct. Mol. Biol* **21**, 1006-1012.

[26] Shimamura, T., Weyand, S., Beckstein, O., Rutherford, N. G., Hadden, J. M., Sharples, D., Sansom, M. S. P., Iwata, S., Henderson, P. J. F. & Cameron, A. D. 2010 Molecular Basis of Alternating Access Membrane Transport by the Sodium-Hydantoin Transporter Mhp1. *Science* **328**, 470-473.

[27] Weyand, S., Shimamura, T., Yajima, S., Suzuki, S. i., Mirza, O., Krusong, K., Carpenter, E. P., Rutherford, N. G., Hadden, J. M., O'Reilly, J., et al. 2008 Structure and Molecular Mechanism of a Nucleobase–Cation–Symport-1 Family Transporter. *Science* **322**, 709-713.

[28] Zomot, E., Gur, M. & Bahar, I. 2015 Microseconds simulations reveal a new sodium-binding site and the mechanism of sodium-coupled substrate uptake by LeuT. *J. Biol. Chem.* **290**, 544-555.

[29] Gur, M., Zomot, E., Cheng, M. H. & Bahar, I. 2015 Energy landscape of LeuT from molecular simulations. *J. Chem. Phys.* **143**, 12B611_611.
